# DNA Polymerase Alpha Subunit B Is a Binding Protein for Erlotinib Resistance in Non-Small Cell Lung Cancer

**DOI:** 10.3390/cancers12092613

**Published:** 2020-09-13

**Authors:** Tae Young Kim, Eun Sun Ji, Ju Yeon Lee, Jin Young Kim, Jong Shin Yoo, A. Marcell Szasz, Balazs Dome, Gyorgy Marko-Varga, Ho Jeong Kwon

**Affiliations:** 1 Chemical Genomics Global Research Lab, Department of Biotechnology, College of Life Science & Biotechnology, Yonsei University, Seoul 120-749, Korea; kty1273@yonsei.ac.kr; 2 Korea Basic Science Institute, Ochang 28119, Korea; eunsunji@kbsi.re.kr (E.S.J.); jylee@kbsi.re.kr (J.Y.L.); jinyoung@kbsi.re.kr (J.Y.K.); jongshin@kbsi.re.kr (J.S.Y.); 3 Department of Tumor Biology, National Korányi Institute of Pulmonology, 1121 Budapest, Hungary; szasz.attila_marcell@med.semmelweis-univ.hu; 4 Department of Bioinformatics, Semmelweis University, 1094 Budapest, Hungary; 5 Division Clinical Protein Science & Imaging, Department of Clinical Sciences (Lund) and Department of Biomedical Engineering, Lund University, SE-221 84 Lund, Sweden; gyorgy.marko-varga@bme.lth.se; 6 Department of Thoracic Surgery, National Institute of Oncology and Semmelweis University, 1117 Budapest, Hungary; dome.balazs@med.semmelweis-univ.hu; 7 Division of Thoracic Surgery, Department of Surgery, Medical University of Vienna, 1090 Vienna, Austria

**Keywords:** Erlotinib, POLA2, DARTS LC-MS/MS, resistance, NSCLC

## Abstract

**Simple Summary:**

Non-small-cell lung carcinoma (NSCLC) covers for almost 85% of all lung cancers and a major contributor to the overall cancer death rate. Erlotinib is used to treat NSCLC via inhibition of epithelial growth factor receptor (EGFR) kinase activity. Despite its high efficacy, recurrence can occur in patients who become resistant to the drug. We performed DARTS LC-MS/MS with SWATH of DIA analysis and identified a novel binding protein of Erlotinib that may underlie NSCLC resistance. Our study indicated that Erlotinib binds POLA2 in addition to EGFR. This was confirmed by DARTS and CETSA results. Importantly, POLA2 expression levels in four NSCLC cell lines were positively correlated with anti-proliferative Erlotinib efficacy (Pearson correlation coefficient, R = 0.9886). These results suggest that POLA2 is a novel complementary target protein of Erlotinib, and could clinically provide validity as a surrogate marker for drug resistance in patients with NSCLC.

**Abstract:**

Erlotinib inhibits epithelial growth factor receptor (EGFR) kinase activity and is used to treat non-small cell lung cancer (NSCLC). Despite its high efficacy, recurrence can occur in patients who become resistant to the drug. To address the underlying mechanism of Erlotinib resistance, we investigated additional mechanisms related to mode-of-drug-action, by multiple protein-binding interactions, besides EGFR by using drug affinity responsive target stability (DARTS) and liquid chromatography-mass spectrometry (LC-MS/MS) methods with non-labeled Erlotinib. DNA polymerase alpha subunit B (POLA2) was identified as a new Erlotinib binding protein that was validated by the DARTS platform, complemented with cellular thermal shift assays. Genetic knock-down of POLA2 promoted the anti-proliferative effect of the drug in the Erlotinib-resistant cell line H1299 with high POLA2 expression, whereas the overexpression of POLA2 restored anti-proliferative effects in the Erlotinib-sensitive cell line HCC827 with low POLA2 expression. Importantly, POLA2 expression levels in four NSCLC cell lines were positively correlated with anti-proliferative Erlotinib efficacy (Pearson correlation coefficient, R = 0.9886). These results suggest that POLA2 is a novel complementary target protein of Erlotinib, and could clinically provide validity as a surrogate marker for drug resistance in patients with NSCLC.

## 1. Introduction

Non-small-cell lung carcinoma (NSCLC) accounts for almost 85% of all lung cancers and is a major contributor to the overall cancer death rate [1]. Lung cancer of the non-small cell histotype is treated with surgical exploration and resection with mediastinal lymph node dissection or systematic lymph node sampling. In case of inoperability, definitive radiotherapy is the choice, supplemented by chemotherapy in case of lymph node involvement. Platinum agents are the backbone of medical treatment, which are accompanied by molecularly driven targeted medications. In case an established histologic subtype and adequate tissue for laboratory testing is present, molecular testing for EGFR, ALK, ROS1, NTRK, and BRAF should be conducted as part of broad molecular profiling [2]. This can be extended to PD-L1 testing as well. The resulting molecular profile drives decision making in thoracic oncology of lung cancer [3].

To treat NSCLC patients, epidermal growth factor receptor (EGFR) is a major drug-able target protein because it is over-expressed in the NSCLC [4]. Gefitinib and Erlotinib were developed as receptor tyrosine kinase inhibitor (RTKi), first-generation EGFR inhibitor. These inhibitors exhibit the outstanding efficacy in EGFR-mutant NSCLC [5,6]. In 2013, Afatinib, a second-generation EGFR inhibitor, was also approved for treating advanced NSCLC patients. It is treated in patients with exon 19 deletions or exon 21 (L858R) substitution mutations, or with advanced squamous cell carcinoma after the failure of platinum-based chemotherapy [7].

Among these inhibitors, Erlotinib was approved in 2004 for all NSCLC patients (wild type EGFR) and in 2014 for advanced NSCLC patients (mutant EGFR) to increase survival in the U.S. Food & Drug Administration (FDA) [8]. After approved by the FDA in 2014, Erlotinib was used in various clinical trials to investigate its efficacy in patients harboring EGFR gene sensitivity mutations (exon 19 deletion or L858R mutation in exon 21) [9,10]. The results showed that patients with mutant EGFR who were treated with RTKi were significantly better off than those treated with traditional chemotherapy [11]. Additionally, in patients with EGFR wild type, it was reported that Erlotinib has similar clinical efficacy compared to chemotherapy in patients with pre-treated advanced NSCLC (wild-EGFR) and no known molecular targetable alterations [12].

Since Erlotinib’s original approval, three generations of EGFR tyrosine kinase inhibitors (TKIs) emerged, all of which possess different pharmacological properties and clinical profiles [3]. Sensitizing EGFR mutation positive patients can be administered preferably Osimertinib, or other agents like Erlotinib, Afatinib, Gefitinib, Dacomitinib, or Erlotinib + Ramucirumab. ALK or ROS1 rearrangement positive patients should get Alectinib, Brigatinib, Ceritinib, or Crizotinib. BRAF V600E mutation bearing patients should receive Dabrafenib + Trametinib or Vemurafenib or Dabrafenib only. Although recent clinical trials have shown better efficacy with second (i.e., Afatinib, Dacomitinib) and third generation (e.g., Osimertinib) TKIs against first-generation TKIs (e.g., Erlotinib), regardless of which treatment is chosen as first-line treatment, resistance with time seems clinically inevitable. Importantly, the availability of subsequent treatment options is an important factor, and the relative advantages of different sequential EGFR-TKI treatments, especially for second and third generation agents, are uncertain. There is currently little such data available to support treatment decisions. Thus, improving survival outcomes is still one of key reasons for using Erlotinib, which has dramatic tumor suppression efficacy.

Plasticity of tumors is a major factor in drug resistance, which can be attributed through the following biological mechanisms, molecular, cellular and pathological levels [13]. Molecular mechanisms include but are not limited to deregulation of genes involved in drug uptake, cell cycle, apoptosis, sphingolipid metabolism and intracellular drug sequestration. At cellular level, stem cells or epithelial–mesenchymal transition may occur, both of which are accompanied by molecular alterations specified above collectively promoting survival advantage and progression of the tumor. Pathological alteration, e.g., switch to small cell lung cancer is a relatively new finding which warrants further investigations, whether neuroendocrine features may come up at a point during the progression of tumors, similarly to that described in prostate cancer [14,15]. Tyrosine-kinase inhibitor treatment results in a selection of those stem cells or clones, which possess an advantageous armamentarium against the given systemic treatment. These cells may use any of the hallmarks identified, making them perfect candidates for survival. Despite these advantages, many patients develop insensitivity to Erlotinib, generally after 9–14 months of treatment [16,17,18,19].

The intrinsic resistance mechanisms have been intensively investigated at the molecular level, which identified a T790M point mutation in the target protein EGFR [20], MET proto-oncogene amplification [21], loss of phosphatase and tensin homologue (PTEN) [22], and epithelial–mesenchymal transition (EMT) [19,23] as relevant resistant mechanisms of the agent.

Other EGFR inhibitors have been associated with different forms of acquired resistance, including human epidermal growth factor receptor 2 (HER2) amplification, AXL receptor tyrosine kinase (AXL) upregulation, mitogen-activated protein kinase (MAPK) 1 amplification, and phosphatidylinositol-4,5-bisphosphate 3-kinase catalytic subunit alpha (PIK3CA) mutation [24]. Interestingly, in a recent report, it was concluded that, beyond EGFR, Erlotinib also induces epigenetic modifications, altered tyrosine kinase activity, and changes within the MAPK and AKT pathways [25]. This indicates that other target proteins besides EGFR could be responsible building up resistance to Erlotinib. Here, we applied drug affinity responsive target stability (DARTS) and liquid chromatography-mass spectrometry (LC-MS/MS) approaches with non-labeled Erlotinib to explore possible binding proteins besides EGFR and discovered that DNA polymerase alpha subunit B (POLA2) positions as a new binding protein candidate, responsible for Erlotinib resistance in NSCLC.

## 2. Results

### 2.1. Selection of an Erlotinib-Resistant Cell Line

**Figure 1:**
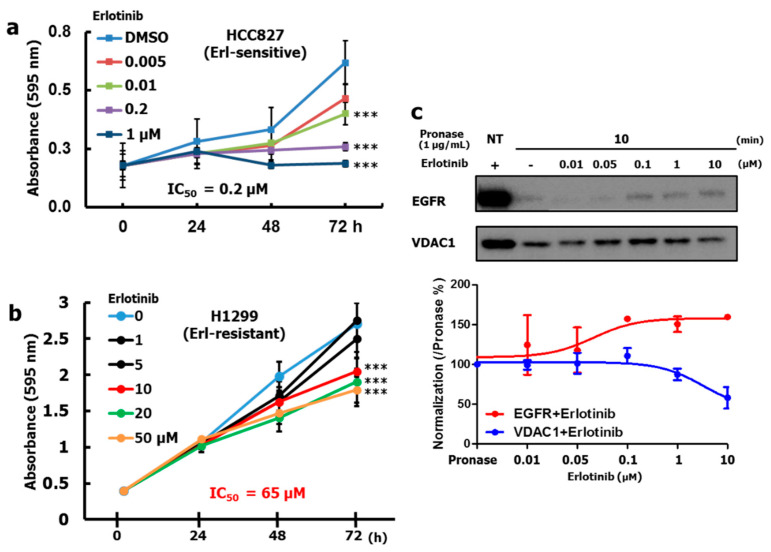
Selection of the Erlotinib-resistant H1299 cell line and Erlotinib binding affinity for its target protein EGFR. (**a**,**b**) anti-proliferative effect of Erlotinib in MTT assays in HCC827 (EGFR exon 19 deletion, Erlotinib-sensitive cell line) and H1299 (EGFR WT, Erlotinib-resistant cell line). *** *p* < 0.001, as determined by Student’s *t*-tests; (**c**) DARTS western blotting demonstrating EGFR binding status to Erlotinib. DARTS analysis for detection of EGFR and VDAC1 as an Erlotinib non-binding protein control. H1299 cells were lysed, diluted to 1 mg/mL, and treated with 0.01~10 μM Erlotinib. Next, pronase was administered at 25 °C over a time course. The graph below the western blot shows the quantification of EGFR (Red) and VDAC1 (Blue) in triplicate experiments. For more details, see Materials & Methods of the main text.

To explore the underlying mechanisms to protein-Erlotinib affinity interactions that builds into a functional drug resistance, we hypothesized the involvement of complementary drug interactions to key regulating NSCLC-proteins, inter-related to the action of EGFR. We therefore attempted to select a NSCLC cell line with Erlotinib resistance. First, the effect of Erlotinib on the proliferation of different cell lines was investigated. The HCC827 cell line was reported to exhibit an Erlotinib-sensitive response due to an EGFR exon 19 deletion mutation. Our results revealed a significant anti-proliferative effect of Erlotinib treatment on HCC827 cells in a dose-dependent manner with a half-maximal inhibitory concentration (IC_50_) of 0.2 µM (Figure 1a). In contrast, the H1299 cell line (EGFR WT) exhibited Erlotinib resistance at IC_50_ 65 µM (Figure 1b). We also investigated if Erlotinib bound EGFR in a H1299 protein pool. To investigate the biophysical interaction between Erlotinib and EGFR, we performed a drug affinity responsive target stability (DARTS) Western blot assay. When samples were pretreated with Erlotinib (before pronase treatment), EGFR stability increased in a time-dependent manner, even under the pronase phase of the assay (Figure S1a). This indicates direct Erlotinib-EGFR binding and may induce conformational changes to confer the pronase resistance. The saturation graph showed stable Erlotinib binding affinity for EGFR in a dose dependency when the Erlotinib concentration was kept at 0.1 µM, implying that Erlotinib tightly interacts with EGFR (Figure 1c).

In silico docking analysis confirmed that Erlotinib specifically binds to LYS 721 and GLU 738 in the ATP binding site of the EGFR wild-type kinase domain (PDB ID: 1M17), which is based on an electronic interaction of van der Waals forces and conventional hydrogen bonds that form a stable energy status (CDOCKER energy: −20.3181) by using Discovery studio^TM^ as predicted [26] (Figure S1b). Notably, these data demonstrate that Erlotinib binds to its known target protein EGFR WT in H1299 cells, but EGFR alone may not be directly responsible for the anti-proliferative activity in this cell line.

### 2.2. DARTS LC-MS/MS SWATH Analysis for Protein Target Identification

**Figure 2:**
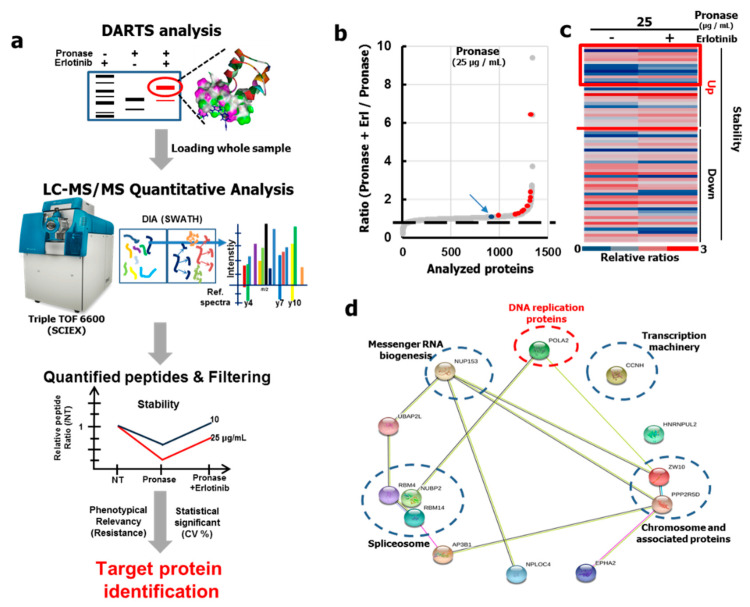
DARTS LC-MS/MS proteome analysis with Erlotinib-treated proteomics using H1299 cell lysates, identifying new Erlotinib binding proteins, responsible for Erlotinib-resistance in H1299 cells. (**a**) Process for the identification of new Erlotinib binding proteins by DARTS LC-MS/MS; (**b**) Identified proteins were analyzed by the averaged quantitative SWATH analysis data from the DARTS sample (pronase 25 µg/mL). A blue dot with an arrow shows EGFR protein, an Erlotinib known target, and red dots are corresponding proteins in Figure S2d. The dashed line is 1 (normalized ratio), which demonstrates the stabilized proteins after Erlotinib treatment; (**c**) The heatmap exhibits relative ratios compared to NT in proteins after pronase treatment with or without Erlotinib. Proteins with ratios greater than 1 were grouped into stability up. The red box area indicates the top 20% of total proteins identified in this study (see Table S2); (**d**) The results of KEGG mapping and STRING bioinformatics analyses for proteins in the red box area of (**c**).

To identify new binding proteins contributing to Erlotinib resistance, we performed DARTS LC-MS/MS sequential window acquisition of all theoretical spectra that would potentially appear by mass spectrometry (SWATH) analysis using H1299 cell lysates as shown in Figure 2a. Before preparing the samples for proteome analysis, we first investigated the optimal DARTS conditions for Erlotinib interactions with EGFR, resulting in a conformational change that ultimately alters the sensitivity upon pronase treatment. In order to enrich the membrane protein fraction, comprising EGFR, we extracted membrane proteins from H1299 cell lysates. The proteome lysate and EGFR expression in each DARTS sample was analyzed and confirmed by western blot analysis. As shown in Figure S2a, EGFR was stabilized by Erlotinib binding with pronase treatment at 10 and 25 μg/mL. These samples were analyzed by SDS gels, where the protein concentration intensity over 10 kDa revealed pronase dose dependence (Figure S2b). In addition, we applied SWATH analysis to verify the DARTS LC-MS/MS output, which acquires a greater pool of target protein than sequencing by data dependent analysis “DDA”. Principal component analysis of the four replicates revealed that the proteome samples clustered according to the ongoing drug impacted disease-biology (Figure S2c), whereby batch effects were eliminated.

### 2.3. Identification of New Erlotinib Binding Proteins

Within the discovery screening phase, we were able to identify 65 proteins that were stabilized or destabilized by the Erlotinib binding (Table S1). Using the ratio graph comparing pronase with Erlotinib treatment versus pronase treatment only, we could verify target protein candidates as red dots, indicating which proteins showed increased stability for both the pronase treatment groups. As EGFR was present in both pronase treatment groups, this would support our feasibility applying the DARTS platform for the identification of Erlotinib binding proteins (as shown in Figure 2b and Figure S2d). We discovered 13 proteins with a 20% increased stability (Figure 2c, Table S2). All of the selected candidate proteins were classified into ten groups by using the Kyoto Encyclopedia of Genes and Genomes (KEGG) mapping, and the Search Tool for the Retrieval of Interacting Genes/Proteins (STRING) database (Figure 2d). Interestingly, many of the binding proteins within group 5 of 10 are functionally known to be localized in the nucleus, and exhibit functions like messenger RNA biogenesis, translation machinery activity, spliceosomal activity, and involvement in DNA replication. Of these functional proteins, POLA2 was selected as a binding protein candidate that affects DNA replication. Previously, Erlotinib was reported to induce EGFR DNA mutations in patients [27], as well as overall DNA mutations in clinically key driver proteins (KRAS, NRAS, EGFR) and other kinase proteins (such as MEK, ERK, MAPK, PI3K, etc.) [25]. We focused on the overall DNA mutations and assumed the phenotype could be linked with POLA2, which plays an essential role in the early stage of chromosomal DNA replication [28].

**Figure 3:**
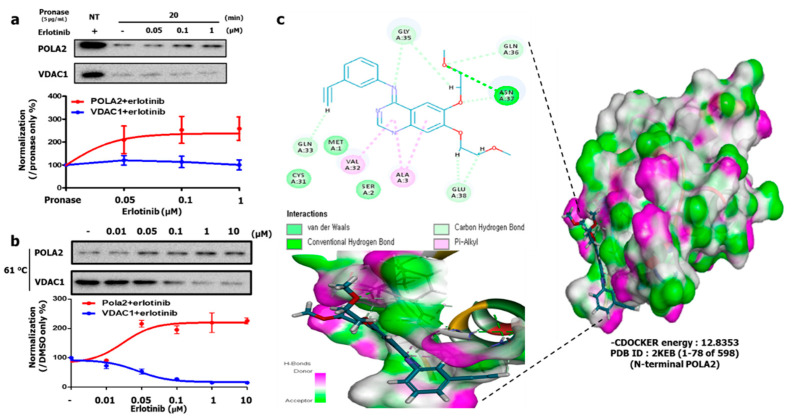
Identification of POLA2 as a new Erlotinib binding protein. (**a**) DARTS assay validating the Erlotinib-POLA2 interaction. POLA2 protein was stabilized in the presence of pronase after Erlotinib treatment in H1299 cells. Pronase treatment was applied for 20 min with Erlotinib in a dose-dependent manner; (**b**) CETSA validation of the erlotinib-POLA2 interaction. POLA2 protein was stabilized under heat treatment (61 °C for 5 min) and different Erlotinib treatments in a dose-dependent manner; (**c**) In silico docking analysis of the interaction between Erlotinib and POLA2 (human N-terminal POLA2, RCSB PDB ID: 2KEB). Erlotinib binds to the TYR34-surrounded POLA2 pocket in the most stable position; binding motifs are depicted as several high-affinity interactions between Erlotinib and the POLA2 pocket. Ligands are shown as gray sticks in charge receptor surfaces. Bonds are shown as dashed lines color-coded as in (**c**).

To investigate whether Erlotinib binds to POLA2, we performed DARTS analysis and western blotting. When the cell lysates samples were pretreated with Erlotinib (before pronase treatment), POLA2 stability significantly increased in a dose- and time-dependent manner, even with pronase treatment (Figure S3a,b). Interestingly, even though proteins were degraded by pronase at 20 min, POLA2 bands were still evident on western blotting, suggesting that direct binding of Erlotinib with POLA2 may induce a conformational change in the protein. Under these conditions, we performed DARTS assays to assess the binding affinity of POLA2 with Erlotinib. As shown in Figure 3a, POLA2 conferred stability with saturation at an Erlotinib level of 0.1 µM, whereas VDAC1 levels were severely decreased by Erlotinib treatment and pronase action, implying that Erlotinib does not bind to VDAC1.

Next, we performed the cellular thermal shift assays (CETSAs) that exploits ligand-induced thermal stabilization of a target protein upon addition of the ligand or compound of interest, confirming a binding event. Compared to the protein alone, a protein-ligand complex is less likely to unfold and subsequently aggregate with increasing temperatures. Based on this concept, cell lysates with and without Erlotinib were incubated at different temperatures followed by centrifugation to separate soluble- (protein-ligand complex) and insoluble-protein (denatured and aggregated). The relationship between aggregation phenomena and its impact by temperature with or without compounds is verified by analyzing the soluble fraction by western blotting [29,30,31]. To investigate optimal conditions for POLA2 aggregation, we first performed temperature-dependent CETSA at 39–67 °C (Figure S3c). POLA2 showed a reproducible intensity of 72% at 61 °C compared to 39 °C. Based on this result, we investigated protein stability at 61 °C with different Erlotinib doses (Figure 3b). Erlotinib thermally stabilized POLA2 from 0.05 μM, and the sigmoidal curve showed a saturated binding pattern starting at 0.1 μM. The DARTS and CETSA data demonstrate that Erlotinib binds to POLA2 with high affinity in the presence of 0.1 μM Erlotinib (Figure 1c).

We conducted in silico docking analyses to validate the interaction between Erlotinib and POLA2. Notably, Erlotinib binds to the N-terminal structure (PDB ID: 2KEB) of POLA2, including the VGLTSEILNSFEHEFLSKRLSKA, the peptide sequence that was identified by SWATH, with a stable energy status (CDOCKER energy: −12.8353) (Figure 3c). Moreover, it interacts with the amino acid sequence stretch; 31~38, excluding a phosphorylation site (Tyr 34) within the POLA2 structure, indicating that Erlotinib occupies the space that blocks POLA2 phosphorylation. Consequently, these data suggest that Erlotinib binds to POLA2 and inhibits its activity. 

### 2.4. POLA2: A Novel Erlotinib Resistance Marker

**Figure 4:**
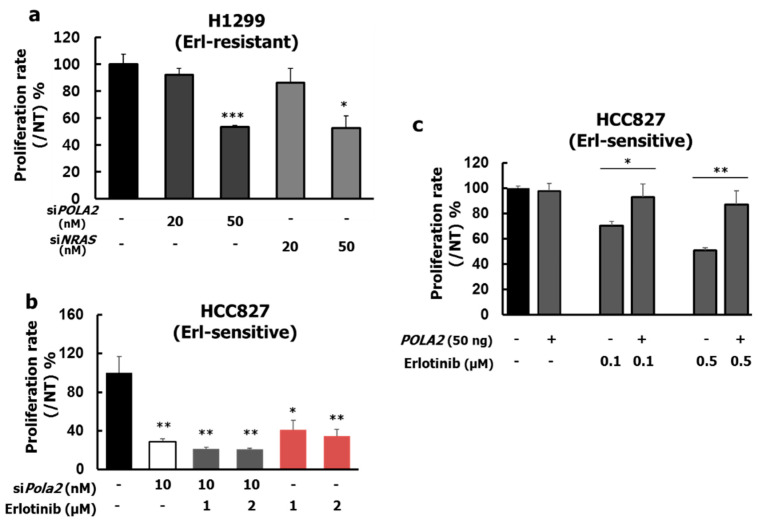
Effects of POLA2 on H1299 proliferation. (**a**) H1299 cells (Erlotinib-resistant line, high POLA2 expression) were transfected with scrambled siRNA, POLA2 siRNA (si*POLA2*), or NRAS siRNA (si*NRAS*) for 96 h. Cells were then used in 3-h MTT assays; (**b**) HCC827 cells (Erlotinib-sensitive line, low POLA2 expression) were transfected with either scrambled siRNA or POLA2 siRNA (si*POLA2*) for 72 h. Cells were then used in 3-h MTS assays; (**c**) POLA2 overexpression rescued the anti-proliferative effect of Erlotinib. Values represent means ± SE; The data presented are the result of four independent experiments. Empty or *POLA2* vectors (50 ng) were transfected into HCC827 cells for 4 h. Erlotinib was given at the indicated concentrations for 72 h. *** *p* < 0.001, ** *p* < 0.01, and * *p* < 0.05 versus control as determined by Student’s *t*-tests.

**Figure 5:**
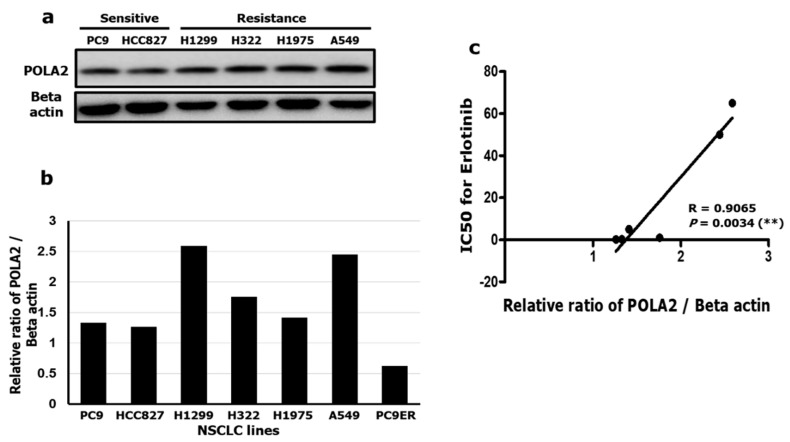
Erlotinib efficacy is POLA2 level dependent. (**a**,**b**) POLA2 expression in Erlotinib-sensitive cell lines (PC9 and HCC827) and Erlotinib-resistant cell lines (H1299 and A549); (**c**) Pearson correlation graph between the IC_50_ of Erlotinib and the relative ratio of POLA2/beta actin in four cell cell lines tested. ** *p* < 0.01 versus control as determined by Student’s *t*-tests.

Based on these results, we performed POLA2 genetic knock-down experiments to explore the protein’s role in H1299 cell proliferation. NRAS was used as a positive control because H1299 cell harbors a driver mutation in the *NRAS* gene [32]. To find the optimal short-interfering RNA (siRNA) concentration, we measured protein levels (Figure S4a,b) and observed a decrease in POLA2 and NRAS at siRNA concentrations of 20 and 50 nM. We assessed cell proliferation effects with both POLA2 siRNA concentrations for 96 h. POLA2 knock-down in H1299 cells achieved a similar anti-proliferative effect with NRAS knock-down treatment (Figure 4a). Interestingly, POLA2 knock-down in HCC827 cells (Figure S4c) exerted a significant anti-proliferative effect of ~80% at 10 nM si*POLA2*, which was higher than for 1 μM Erlotinib (Figure 4b). These results may be due to differences in POLA2 expression levels in H1299 and HCC827 cells (Figure 5a).

Based on the low endogenous POLA2 expression in HCC827 cells, we assessed the proliferative effect after transfection of plasmid DNA including POLA2 to confirm whether it could be a useful Erlotinib resistance marker (Figure S4d). Enhancing POLA2 protein expression recovered the anti-proliferative effect of Erlotinib in a dose-dependent fashion, with 22.5% and 36.1% increases compared with treatment with Erlotinib 0.1 and 0.5 μM alone, respectively (Figure 4c). These data demonstrate that POLA2 expression might contribute to Erlotinib resistance.

### 2.5. Pola2 Is a Potential Erlotinib-Resistant Biomarker

To further investigate whether POLA2 could be a potential resistance biomarker in NSCLC, its expression levels were examined in four NSCLC cell lines; PC9 and HCC827, Erlotinib-sensitive cell lines; H1299 and A549, Erlotinib-resistant cell lines, to assess the correlation between Erlotinib and POLA2 levels (Figure 5a). The expression levels of POLA2 were higher in Erlotinib-resistant cell lines compared to sensitive cell lines (Figure 5b). Next, the IC_50_ values (Table S3) were determined in cell proliferation assays with all four-cell lines (Figure 1a,b and Figure S5a,b). Pearson correlation coefficients were calculated to reveal the relationship between POLA2 expression and Erlotinib efficacy. We observed a strong positive correlation (R = 0.9886) between POLA2 expression level and the IC_50_ of Erlotinib (Figure 5c). These results demonstrated that POLA2 could be a potential Erlotinib resistance biomarker in NSCLC.

## 3. Discussion

In this study, we used DARTS and CETSA methods to identify POLA2 as a novel Erlotinib binding protein responsible underlying drug resistance in NSCLC cells. These approaches utilize the concept that protein stability is influenced by high-affinity ligand interactions. Although we have provided the evidences that POLA2 contributes to Erlotinib resistance, it remains unclear how POLA2 induces DNA mutations. Because POLA2 is a key component of the polymerase alpha complex and plays the role of primase, Erlotinib may interrupt to synthesize a correct DNA primer in front of Okazaki fragments. Similarly, Pavlov et al. reported that DNA mutations were generated by functional suppression of DNA polymerase alpha (POLA1) with base substitution [33]. Although the proofreading activities of polymerase delta and epsilon are effective in repairing DNA sequences [34], inhibition of POLA2 by long-term Erlotinib treatment may contribute to increase in abnormal DNA sequences. This could partially explain a case effort to make Erlotinib-sensitive HCC827 to Erlotinib-resistant HCC827 cell line [35,36,37].

Erlotinib is a tyrosine kinase inhibitor (TKI) and has a quinazoline scaffold structure. Similarly, Gefitinib and Afatinib are analogue compounds with quinazoline scaffold structures that suppress EGFR signaling [38]. It was recently reported that EGFR inhibitors lead to acquired resistance by inducing DNA mutations (including gain-of-function changes) to accelerate onco-genesis [24]. Based on the functional and structural similarities of these compounds, they might also interact with the POLA2 protein to induce acquired resistance and cause DNA mutations. These mechanistic aspects are under investigation.

In the clinical setting, upon advanced stage NSCLC, Erlotinib treatment is administered, where as a consequence to drug impact, resistance develops over time [16,17,18,19]. A decision should be made to switch from first generation TKI to second, and later to third generation agents [3]. However, an Erlotinib re-challenge drug concept may also come under consideration. Our study highlights the importance of testing for the activities of mode-of-drug impacting proteins, such as POLA2 protein.

In this study, DARTS LC-MS/MS SWATH analysis was used to identify a number of binding protein candidates of Erlotinib [39,40,41]. EGFR, which is a known target of Erlotinib was also identified in this SWATH analysis. Besides EGFR, the screen revealed 10 binding protein candidates. Of these, EPHA2 was previously reported as a protein underlying Erlotinib resistance. EPHA2 inhibition was validated to recover anti-tumor efficacy in Erlotinib-resistant tumors [42]. Based on this, POLA2 could be targeted to improve Erlotinib efficacy, and the 10 target candidates are worth studying in future investigations of Erlotinib-resistant proteins.

## 4. Materials and Methods

### 4.1. Materials

Erlotinib (N-(3-ethynyl phenyl)-6, 7-bis (2-methoxyethoxy)-4-quinazolinamine, C22H23N3O4, ≥98%) was purchased from Cayman Chemical (Ann Arbor, MI, USA). Trifluoroacetic acid and methanol were purchased from Sigma-Aldrich (St Louis, MO, USA). Acetonitrile and acetic acid (hyper-grade for LC-MS) were purchased from Merck (Darmstadt, Germany). Anti-EGFR (#4267, 1:3000) was purchased from Cell Signaling Technology (Danvers, MA, USA). anti-β-tubulin (ab15568, 1:3000) and VDAC1 (ab154856, 1:3000) antibodies were purchased from Abcam, Cambridge, UK. Anti-POLA2 antibodies were from two companies (ab103591, 1:2000, Abcam, Cambridge, UK; #TA807639, 1:2000, Origene Technologies, Rockville, MD, USA), and anti-NRAS was from Invitrogen (#PA5-28861; 1:1000, Invitrogen, Carlsbad, CA, USA). Matrigel was purchased from Corning (Cat. No. 354234, Corning, New York, NY, USA).

### 4.2. Cell Culture

Dr. Ho-Young Lee (College of Pharmacy, Seoul National University, Seoul, Korea) kindly provided human NSCLC cell lines (PC9, HCC827, H1299, A549). These cell lines were maintained in RPMI1640 medium supplemented with 10% fetal bovine serum (Life Technologies, Gaithersburg, MD, USA). All cell lines were maintained at 37 °C in a humidified incubator with 5% CO_2_ and media pH 7.4.

### 4.3. Proliferation Assay

Cell proliferation was assessed with (3-(4,5-dimethylthiazol-2-yl)-2,5-diphenyltetrazolium bromide kits (MTT, VWR Life Science, Radnor, PA, USA) and MTS cell proliferation kits (ab197010, Abcam). HCC827 and H1299 cells were seeded in 96-well plates at densities of 3 × 10^3^ and 2 × 10^3^ cells/well, respectively. Cells were incubated overnight and treated with the compounds for 24 to 72 h. Then, 25 μL of MTT (2 mg/mL) in PBS or MTS solution were added for 3 h. Cell proliferation was assessed by measuring absorbance at 595 nm (MTT) and 490 nm (MTS) on a Victor 5 multi-label plate reader (Perkin Elmer, Waltham, MA, USA).

### 4.4. DARTS Assay

DARTS is a label-free method, which means that it does not require chemical modifications of the protein or drug. It is based on the concept that ligand-bound proteins show altered stability against proteolysis compared to ligand-unbound proteins. Indeed, a target protein bound to its ligand shows increased structural stability that can be detected as an augmented protein band on SDS-polyacrylamide gel electrophoresis (PAGE) compared with the ligand-free protein [30].

H1299 cells were scraped and lysed with the Mem-PER Plus membrane protein extraction kit (Cat. No. 89842, Thermo Fisher Scientific, Waltham, MA, USA). Following protein quantification using the bicinchoninic acid (BCA) assays, samples were diluted to a protein concentration of 1 mg/mL for western blotting and of 5 mg/mL for LC-MS/MS analysis. To determine the drug-protein binding interaction, samples were incubated with similar concentration of Erlotinib with in vitro level at 4 °C for 4 h. Samples were then treated with pronase (Roche, Basal, Switzerland) for proteolysis at 25 °C. After digestion with Pronase, aliquots of samples were mixed with SDS and boiled.

To prepare the cell lysate, H1299 cells were scraped and lysed with the Mem-PER Plus membrane protein extraction kit (Cat. No. 89842, Thermo Fisher Scientific). Following quantification of membrane proteins using BCA assays, samples were diluted to a protein concentration of 1 mg/mL. All buffers were supplemented with Halt™ Protease Inhibitor Cocktail (100X)(Cat. No. 78429, Thermo Fisher Scientific) diluted to a final concentration of 2X.

After 10-min incubation at room temperature, the respective lysates were divided into 100-μL aliquots and heated at different temperatures for 5 min in a heat block (Thermo BATH ALB64, PINEPCR) followed by cooling for 5 min at room temperature. The heated lysates were centrifuged at 20,000× *g* for 20 min at 4 °C to separate the soluble fractions from precipitates. The supernatants were transferred to new micro-tubes and analyzed by SDS-PAGE and western blot.

### 4.5. In Silico Docking Study

All molecular docking analyses were performed with Discovery Studio 2018 software (v18.1.0.17334, Accelrys, San Diego, CA, USA) adopting the CHARMm force field. The crystal structure of the human EGFR kinase domain (PDB ID 1M17) and N-terminal POLA2 (PDB ID 2KEB) were extracted from the RCSB protein data bank.

The protein structures of the nucleotide-binding domain of POLA2 were energy minimized using the Powell algorithm. The ligands were docked using Ligandfit, and the parameters were validated using the ligand from both PDB IDs (1M17 and 2KEB) including crystal structures.

### 4.6. Immunoblot Analysis

Cell lysates were separated by 8%, 10%, or 12.5% SDS-PAGE and transferred to polyvinylidene fluoride membranes (Millipore, Billerica, MA, USA) using standard electroblotting procedures and the Trans-Blot SD Semi-Dry transfer system (Bio-Rad, Hercules, CA, USA). Blots were blocked and immunolabeled overnight at 4°C with primary antibodies. Immunolabeling was detected with an enhanced chemiluminescence kit (Bio-Rad) according to the manufacturer’s instructions. Images were quantified with Image Lab^™^ software (Bio-Rad). β-actin was used as an internal control. Full bolts can be found at Figures S6 and S7.

### 4.7. RNA Interference and Overexpression Analysis

Human POLA2-specific siRNA (siPOLA2) was purchased from GE Healthcare Dharmacon (Lafayette, CO, USA). The ON-TARGETplus/SMARTpool-derived siRNA against human POLA2 mRNA was designated as L-016027-00-0005. The target mRNA sequences of this siRNA were J-016027-05 (5′-UGA GAG AUG UGC ACC AUG A-3′), J-016027-06 (5′-GCG AGG CUC UAA UUG A -3′), J-01627-07 (5′-GGA AAC UUG CCA A-3′), and J-016027-08 (5′-ACA CAU AAA GUU GGC CUU A-3′). The ON-TARGETplus/SMARTpool-derived siRNA against human NRAS mRNA was designated as L-003919-00-0005. The target mRNA sequences of this siRNA were J-003919-05 (5′-GAG CAG AUU AAG CGA GUA A-3′), J-003919-06 (5′-GAA AUA CGC CAG UAC CGA A-3′), J-003919-07 (5′-GUG AUG UAA CAA GAU A-3′), and J-003919-08 (5′-GCA CUG ACA AUC CAG CUA A-3′). For genetic knockdown, H1299 cells and HCC827 cells were transfected with scrambled, or POLA2 siRNA using Lipofectamine RNAiMAX transfection reagent and Opti-MEM (both from Invitrogen) according to the manufacturer’s instructions.

Human POLA2 cDNA clone (Origene Technologies) was purchased as pCMV6-MYC-DKK-POLA2. For POLA2 overexpression, H1299 was transfected with scrambled or pCMV6-MYC-DKK-POLA2 using Lipofectamine LTX transfection reagent and Opti-MEM (both Invitrogen) according to the manufacturer’s instructions.

### 4.8. Statistical Analysis

Results are shown as mean ± standard error, and all statistical analyses were calculated with GraphPad Prism (v. 5.00 for Windows, GraphPad Software, San Diego, CA, USA). The values were taken in coefficient of variation <30%. Student’s *t*-tests were applied to identify significant differences between the control and test groups. A *p*-value < 0.05 was considered statistically significant.

Correlations between two parameters were determined using the Pearson correlation coefficient value (R), which was considered significant at *p* < 0.05 on pairwise *t*-tests.

## 5. Conclusions

We performed DARTS LC-MS/MS with SWATH of DIA analysis and identified a novel binding protein of Erlotinib that may underlie NSCLC resistance. This method takes advantage of an un-modified compound to rapidly sort out target candidates with the auto-quantify program of SWATH analysis. This powerful strategy indicated that Erlotinib binds POLA2 in addition to EGFR. This was confirmed by DARTS and CETSA results. Both methods also revealed that Erlotinib has the same binding affinity for POLA2 and EGFR (0.1 μM).

We confirmed proliferation suppression by genetic knock-down of POLA2. Overexpression of POLA2 in HCC827 cells, which normally express low levels of POLA2, ameliorated the anti-proliferative effect of Erlotinib and led to drug resistance. Consistent with this result, POLA2 levels in four NSCLC cell lines were strongly correlated (R = 0.9886) with an increased IC_50_ for Erlotinib. This result indicated that POLA2 could be a potential biomarker of Erlotinib resistance in NSCLC.

In the era of molecular oncologicy, NGS is widely applied, and as proteomics applications emerge, POLA2 may be included in a targeted screening for resistance mechanisms against TKIs, as this or other such alterations may be found in progressive tumors in any clinical setting where drug sensitivity decreases.

## Supplementary Materials

The additional resulting data, figures and read-outs are available online at https://www.mdpi.com/2072-6694/12/9/2613/s1, Figure S1: Characterization of EGFR as an Erlotinib target, Figure S2: SDS-PAGE analysis of the H1299 proteome pool visualized using CBB staining, Figure S3: Characterization of POLA2 as a novel Erlotinib target and the optimal concentration, Figure S4: Genetic knockdown of POLA2 and NRAS in H1299 and HCC827 cells, Figure S5: Proliferative effect of Erlotinib on 72-h MTT assays, Figure S6: The full blots for all of western blot used in main figures, Figure S7: The full blots for all of western blot used in supplementary figures, Table S1: Criteria to select the target candidates of Erlotinib, Table S2: List of the target candidates (red box in Figure 2c), Table S3: IC_50_ values for Erlotinib in NSCLC cell lines.
