# Supplementary material for: DNA Polymerase Alpha Subunit B Is a Binding Protein for Erlotinib Resistance in Non-Small Cell Lung Cancer
Source: Cancers (Basel). 2020 Sep 13;12(9):2613. doi: 10.3390/cancers12092613 (PMC7564424; doi:10.3390/cancers12092613)
Supplement: Supplementary file 1 [file cancers-12-02613-s001.pdf]

Supplementary Materials: DNA Polymerase Alpha Subunit B Is a Binding Protein for Erlotinib Resistance in Non-Small Cell Lung Cancer

Tae Young Kim, Eun Sun Ji, Ju Yeon Lee, Jin Young Kim, Jong Shin Yoo, A. Marcell Szasz, Balazs Dome, Gyorgy Marko-Varga and Ho Jeong Kwon

Supplementary Materials and Methods:

LC-MS/MS Sample Preparation

For each sample, DTT was added to a final concentration of 10 mM, and the samples were then incubated for 30 min at room temperature, with agitation. Next, IAA was added to each sample to a final concentration of 10 mM, and each sample was then placed in the dark for 30 min at room temperature. Subsequently, protein samples were digested into peptides using trypsin; these peptides were incubated with end-over-end rotation at 37 °C for 4 h. The final solutions were dried using a SpeedVac, and the dried peptides were re-dissolved in an aqueous solution of 0.1% formic acid prior to quantification.

Proteome Analysis

We referenced previous publication [8]. LC–MS/MS Analysis: IDA (information-dependent acquisition) and SWATH (sequential windowed acquisition of all theoretical fragment-ion spectra) mass spectrum techniques were used to acquire tandem MS data on a Triple TOF 6600 tandem mass spectrometer (AB Sciex, Concord, Ontario, Canada) coupled to an Ekspert nanoLC 400 system (AB Sciex). An autosampler was used to load individual 5 μL aliquots of the peptide solutions into a C18 trap column of inner diameter (i.d.) 180 μm, length 20 mm, and particle size 5 μm (Waters, Milford, MA, USA). Peptides were desalted and concentrated on the trap column for 10 min at a flow rate of 5 μL/ min. Trapped peptides were then back-flushed and separated on a homemade microcapillary C18 column of i.d. 100 μm and length 200 mm (Reprosil Pur 120, C18-AQ, particle size 3 μm) at a flow rate of 300 nL/min. The mobile phases were composed of 100% water (A) and 100% acetonitrile (ACN) (B), each containing 0.1% formic acid. The liquid chromatography (LC) gradient began with 5% mobile phase B and ramped to 10% mobile phase B, for 1 min. While, 10% mobile phase B was maintained for 4 min, 10% mobile phase B was linearly ramped to 40% for 90 min and then to 95% for 1 min. Then, 95% mobile phase B was maintained for 13 min before descending to 5% B for another 1 min. The column was re-equilibrated with 5% B for 10 min before the next run. The voltage of the nanoelectrospray was 2.0 kV. During the chromatographic separation, triple time-of-flight mass spectrometry (TOF-MS) was performed using either an IDA MS or a SWATH MS. In IDA mode, a TOF-MS survey scan was acquired at m/z 400–2000 with 250 ms accumulation time. The 10 most intense precursor ions (2+–5+) in the survey scan were consecutively isolated for product ion scans. Dynamic exclusion was used for 45 s. Product ion spectra were accumulated for 100 ms in the mass range m/z 100–2000, with rolling collision energy.

IDA DATA-Based SWATH Library Generation: SWATH data were acquired three times for each sample. In SWATH mode, TOF-MS survey scans were acquired (m/z 400–2000, 250 ms); this was followed by 34 product ion scans, with consecutive constant Q1 windows (25 Da) in the mass range m/z 400–1250. Product ion spectra were accumulated for 49 ms in the mass range m/z 100–2000, with rolling collision energy. IDA Data-Based SWATH Library Generation. IDA MS/ MS data were subjected to database searches by ProteinPilot (V5.0, AB Sciex, Framingham, MA, USA) using the Paragon algorithm. The UniProt human database (August 2014 version, www.uniprot.org) was used for database searches. Search parameters were as follows. Sample type: identification. Cys alkylation: iodoacetamide. Digestion: trypsin. Special factors: none. ID focus: allow biological modifications. The

group file from the database search was loaded to PeakView (V2.2 with SWATH Quantitation plug-in, AB Sciex, Framingham, MA, USA) and exported as libraries in CSV format.

SWATH Peak Extraction: PeakView V2.2 with SWATH quantitation plug-in (AB Sciex) was used to extract SWATH MS peak areas using the library generated in our study. PeakView uses a set of processing settings to filter the ion library and determine which peptides or transitions should be used for quantification. These settings include: the maximum number of peptides (50) per protein to be included from the imported ion library; the number of transitions (three) per peptide; a peptide confidence threshold (95%) in percentage that is used to remove the peptides with a confidence below that value; a false discovery rate (FDR < 1%) in percentage, which is used to filter the SWATH extraction results and only export the peptide peak groups displaying a false positive rate below that value; and XIC (Extracted Ion Chromatogram) retention time window (15 min) and mass tolerance window (50 ppm) for RT and m/z tolerance, to pick the transitions. After SWATH peak extraction, the transition ion peak areas, peptide peak areas, and protein peak areas were exported in Excel format for further statistical analysis. The ion peak areas exported by PeakView for each sample were normalized, by total area normalization, using MarkerView (V.1.2.1, AB Sciex, Framingham, MA, USA). Specifically, the sum total of all ion intensities for each sample was calculated, and the maximum of those totals was determined. The ion intensities of each sample were divided by the ratio of [total ion intensities]/[maximum total ion intensity], ensuring that the normalized ion intensities sum to the same value for all samples. Normalized protein peak areas were automatically performed via PCA (principal component analysis) and then grouped by PC variance in MarkerView; 25 groups were generated. These results were then exported into Excel format according to peptide and protein, which we refer to henceforth as peptide peak area and protein peak area.

Finally, the results of proteins showing significant changes in expression level were checked by manual validation, and their peak areas were recalculated based solely on reliable peptide peaks acquired during SWATH extraction.

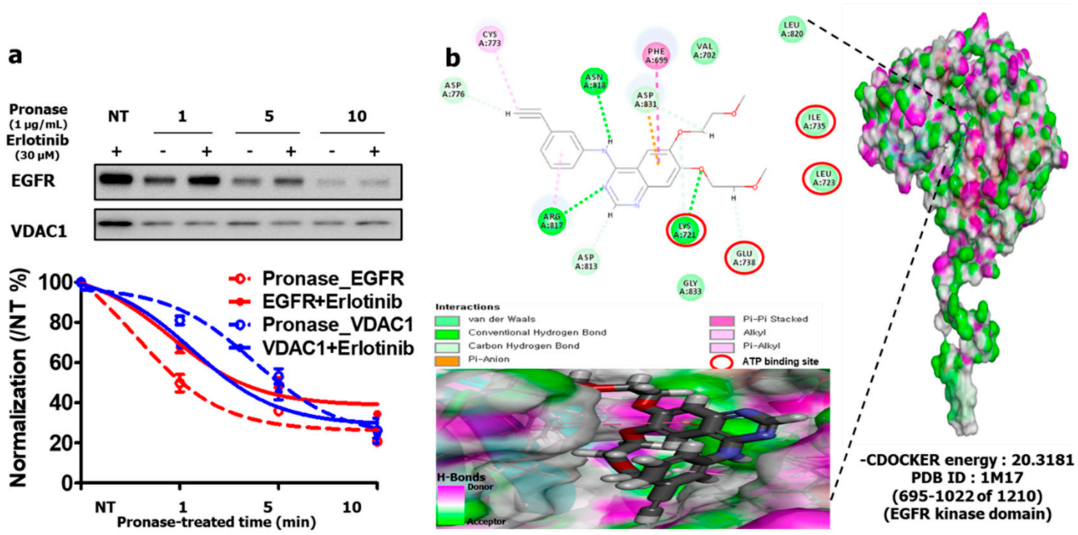

Figure S1. Characterization of EGFR as an Erlotinib target. (**a**) Optimal condition of DARTS assay for target validation. EGFR protein was stabilized upon Pronase treatment compared with destabilization of VDAC1 in H1299 cell lysates. The sigmoidal curve shows the quantitative data; (**b**) In silico docking model of Erlotinib interacting with EGFR (Human EGFR kinase domain, RCSB PDB ID: 1M17). Erlotinib binds to amino acid LYS 721, GLU 738, ILE 735, and LEU 723 of ATP binding site (Red circles) with van der Waals force and hydrogen bond (Up panel). Ligands are shown as bold sticks in displayed charge receptor surfaces. Bonds are shown as dashed lines color-coded as shown in that picture (Down panel).

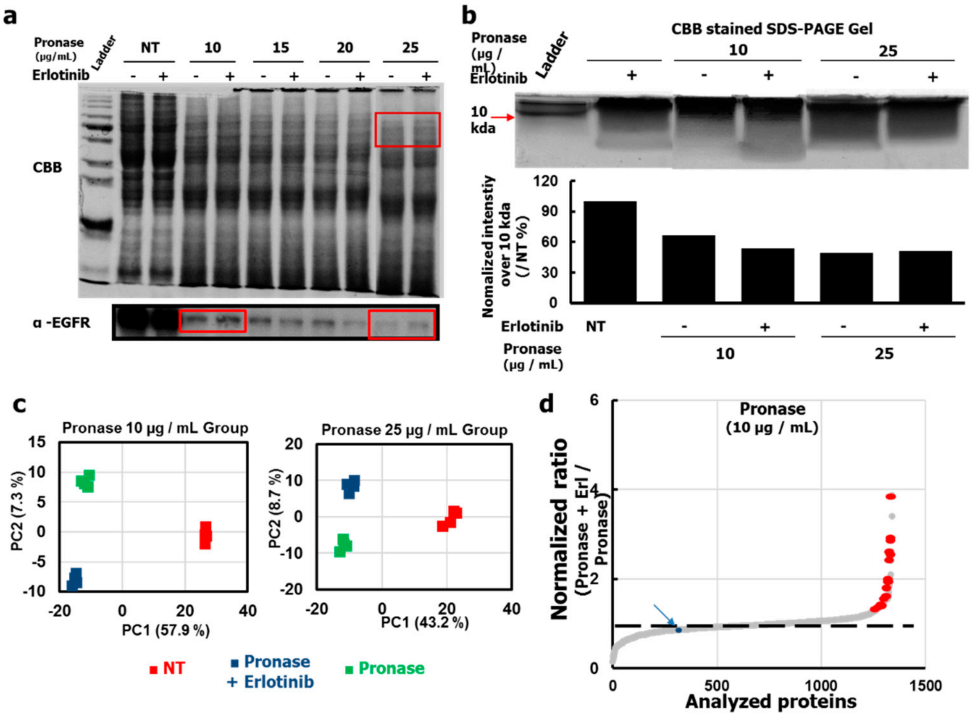

Figure S2. SDS-PAGE analysis of the H1299 proteome pool visualized using CBB staining. H1299 cells were lysed and diluted to 5 mg/mL. After dilution, these cells were treated in 30 μM Erlotinib and incubated at 4 °C for 4 h. After the drug treatment, Pronase treatment was administered at 25 °C for 15 min. After digestion with Pronase, aliquots of samples were mixed with SDS and boiled. (**a**) This samples were loaded in CBB stained 8% SDS-PAGE gel. 10 differential protein bands were analyzed by image analysis of Pronase dose dependent (10 ~ 25 μg/mL) with Erlotinib 30 μM treatment and confirmed EGFR expression in same sample; (**b**) Sample preparation for DARTS LC-MS/MS analysis. CBB stained 15% SDS-PAGE gel. It was loaded with remaining sample after demonstrating the proteome change in Figure S2a. The graph shows intensity data over 10 kda normalized by NT levels; (**c**) The proteome status of DARTS LC-MS/MS samples measured in 4 replicate segregated into NT (Red), Pronase only (Blue), and Pronase with Erlotinib (Green) subgroup by PCA analysis; (**d**) Identified proteins were arranged by quantitative SWATH analysis data from the DARTS sample (Pronase 10 μg/mL). Blue dot with an arrow means EGFR protein and red dots are coincided with protein in Figure 2b. Dash line is 1 of normalized ratio, which means increased quantitatively proteins by Erlotinib.

Table S1. Criteria to select the target candidates of Erlotinib.

Criteria	Group.	
	P10	P25
Total proteins	1594	1762
Common Proteins	1402 (+EGFR)	
Correlation of Ratio (Pronase + Erlotinib / Pronase only) >1	71	
Exclusion of over CV 50%	65	
Top 20%	13	

Table S2. List of the target candidates (red box of main figure 2c).

Protein Description	Relationship with Resistance
DNA polymerase alpha subunit B OS = Homo sapiens GN = POLA2 PE = 1 SV = 2	+++
Ephrin type-A receptor 2 OS = Homo sapiens GN = EPHA2 PE = 1 SV = 2	+++
RNA-binding protein 14 OS = Homo sapiens GN = RBM14 PE = 1 SV = 2	+++
Nuclear protein localization protein 4 homolog OS = Homo sapiens GN = NPLOC4 PE = 1 SV = 3	–
Heterogeneous nuclear ribonucleoprotein U-like protein 2 OS = Homo sapiens GN = HNRNPUL2 PE = 1 SV = 1	–
Ubiquitin-associated protein 2-like OS = Homo sapiens GN = UBAP2L PE = 1 SV = 2	–
Cyclin-H OS = Homo sapiens GN = CCNH PE = 1 SV = 1	–
Nuclear pore complex protein Nup153 OS = Homo sapiens GN = NUP153 PE = 1 SV = 2	–
Cytosolic Fe-S cluster assembly factor NUBP2 OS = Homo sapiens GN = NUBP2 PE = 1 SV = 1	–
AP-3 complex subunit beta-1 OS = Homo sapiens GN = AP3B1 PE = 1 SV = 3	–
Serine/threonine-protein phosphatase 2A 56 kDa regulatory subunit delta isoform OS = Homo sapiens GN = PPP2R5D PE = 1 SV = 2	–
Centromere/kinetochore protein zw10 homolog OS = Homo sapiens GN = ZW10 PE = 1 SV = 3	–
RNA-binding protein 4 OS = Homo sapiens GN = RBM4 PE = 1 SV = 1	–

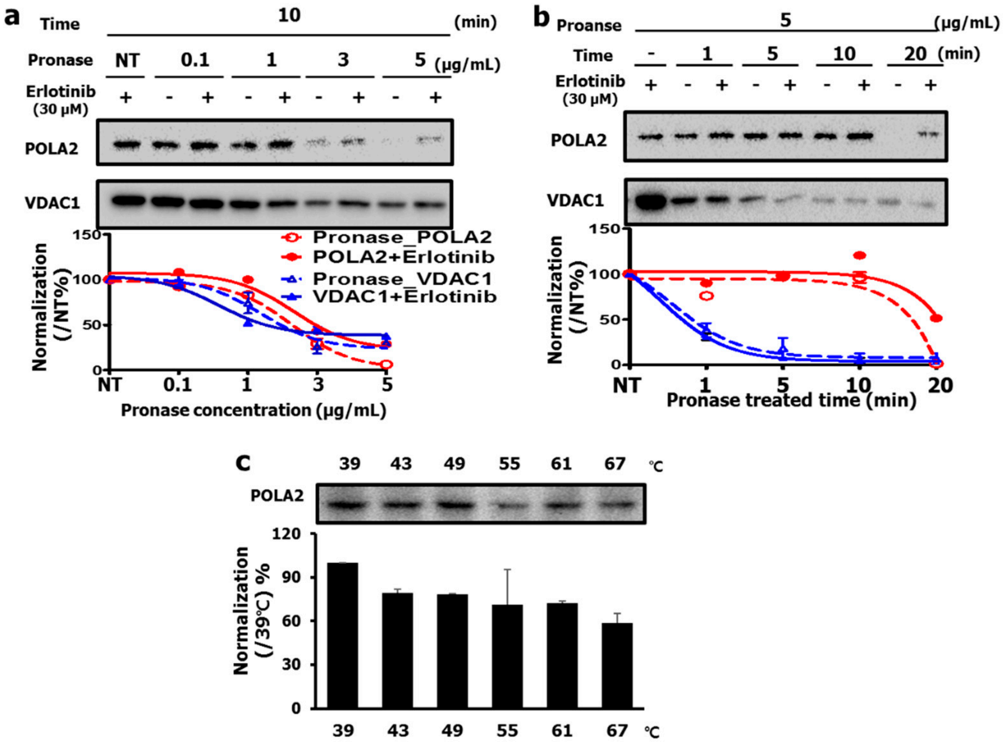

Figure S3. Characterization of POLA2 as a novel Erlotinib target and the optimal concentration. DARTS assays were performed by Pronase-dose (**a**) and time dependent (**b**); (**c**) The CETSA assay demonstrated the intensity of POLA2 on temperature-dependent (39 ~ 67 °C) for 5 min. More details of western blot, please view at the material & method. Values in this graph represent mean ± SEM from three independent experiments.

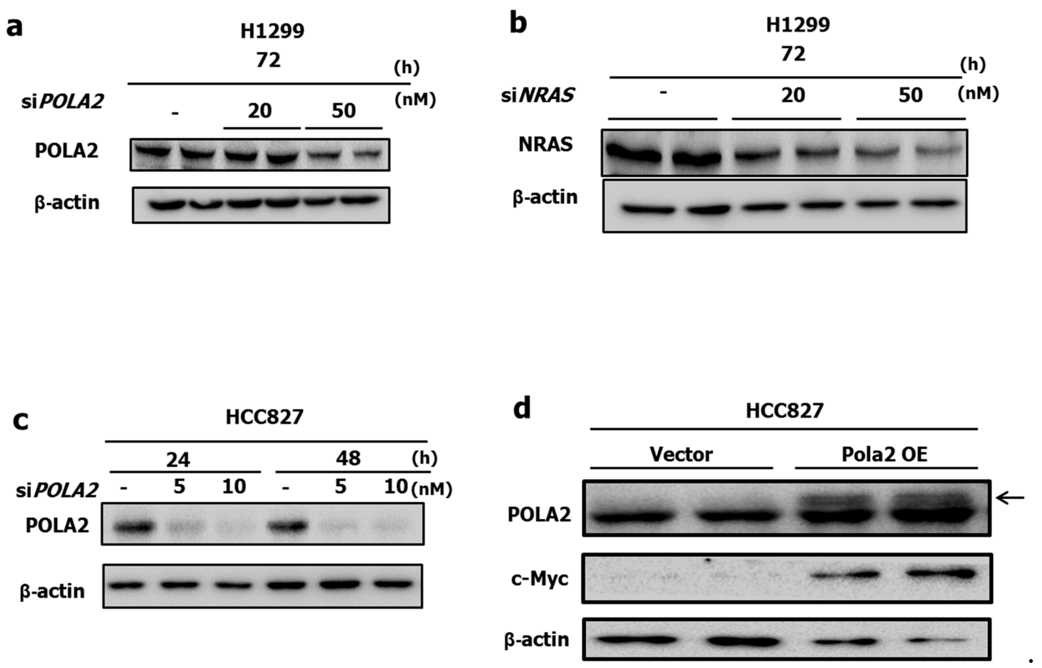

Figure S4. Genetic knockdown of POLA2 and NRAS in H1299 and HCC827 cells. (**a**) si*POLA2* 20 and 50 nM were transfected in H1299 for 72 h; (**b**) si*NRAS* 20 and 50 nM were transfected in H1299 for 72 h; (**c**) Genetic knockdown of POLA2 by siRNA in HCC827 cell line at time (24, 48 h) and dose (5, 10 nM) response; (**d**) Overexpression of POLA2 in HCC827. Vector and *POLA2* were transfected in HCC827. Indicated arrow means shifted bands by tagged POLA2 with c-myc and DDK. Expression of Beta-actin was detected to use to inner control.

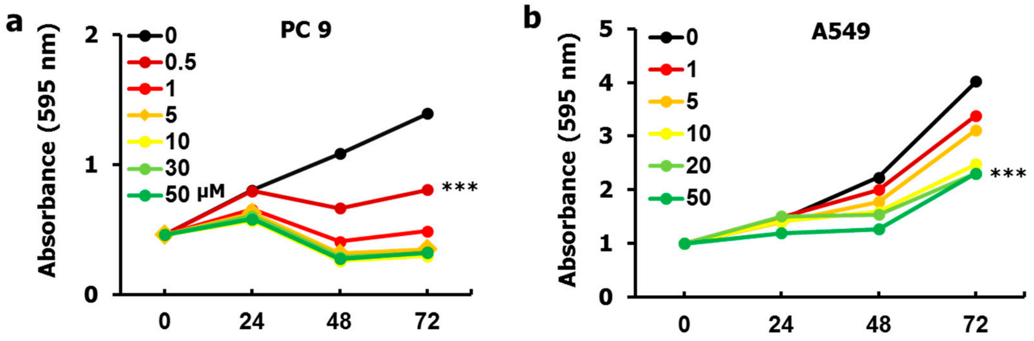

Figure S5. Proliferative effect of Erlotinib on 72-h MTT assays. NSCLC cell lines (**a**) PC9 and (**b**) A549 were measured absorbance (595 nm) by plate reader. Values in this graph represent means from three independent experiments.

Table S3. IC_{50} values for Erlotinib on NSCLC cell lines.

Cell line	PC9	HCC827	H1299
IC_{50} (μM)	0.3	0.2	65

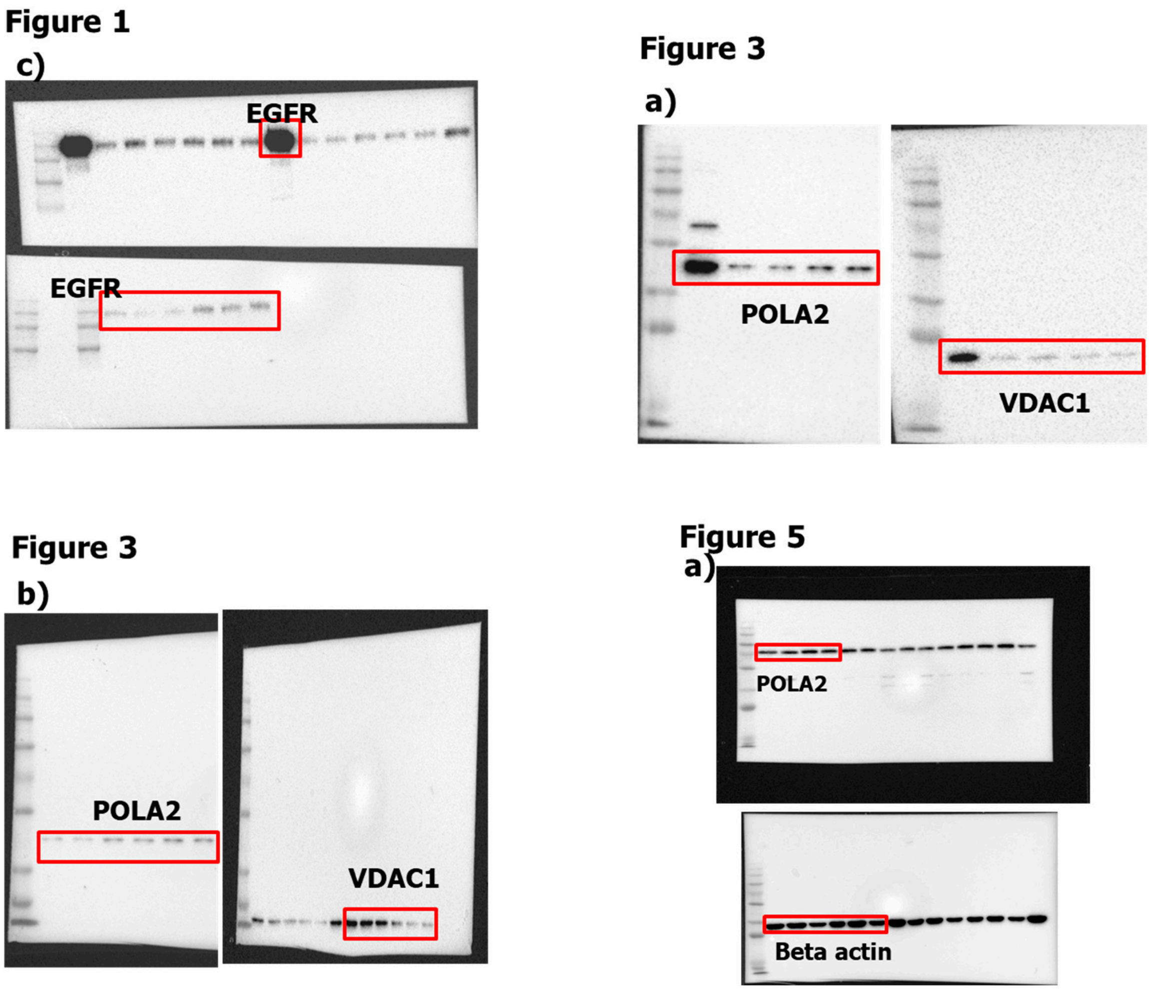

Figure S6. The full blots for all of western blot used in main figures.

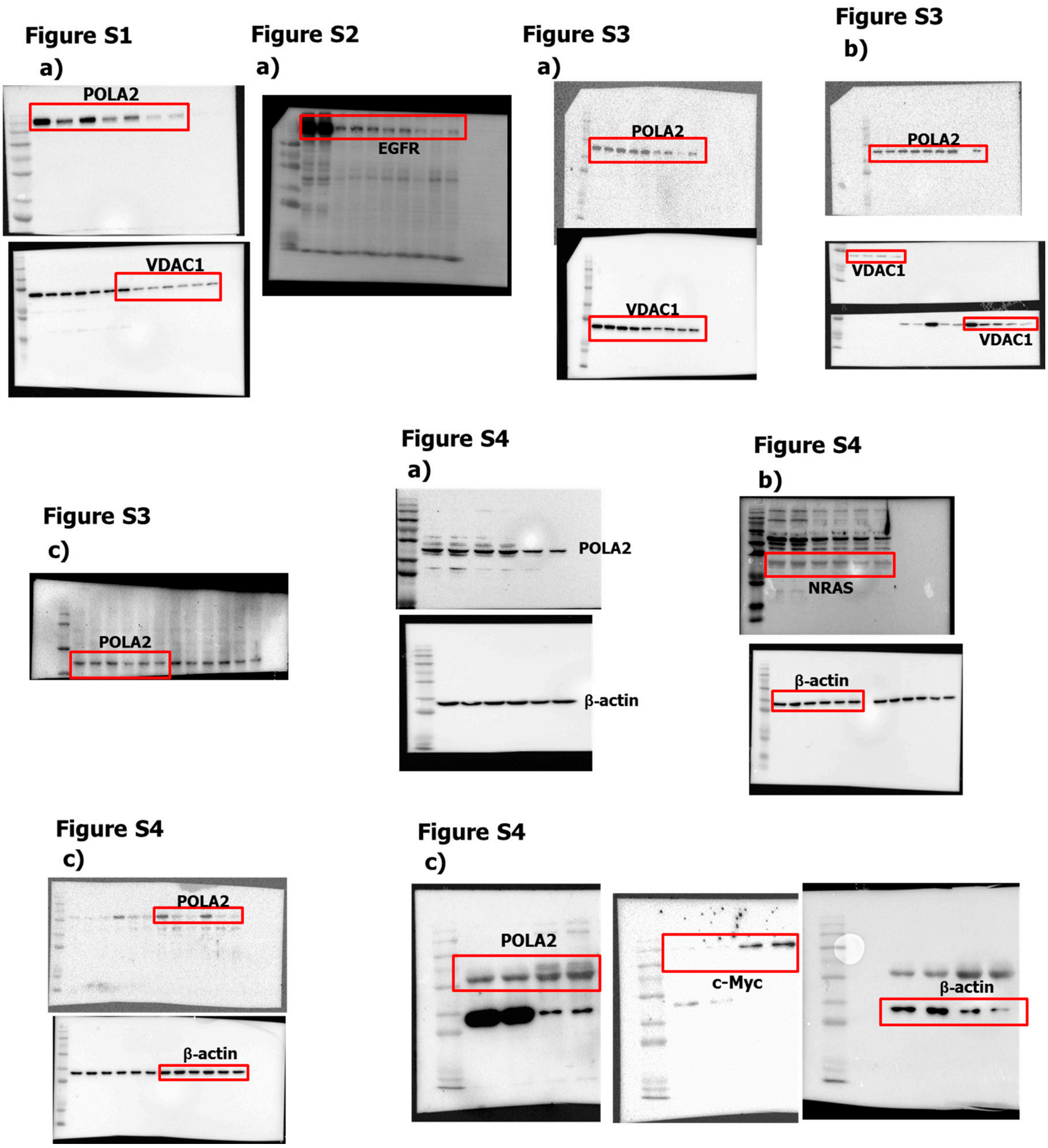

Figure S7. The full blots for all of western blot used in supplementary figures.

© 2020 by the authors. Licensee MDPI, Basel, Switzerland. This article is an open access article distributed under the terms and conditions of the Creative Commons Attribution (CC BY) license (http://creativecommons.org/licenses/by/4.0/).
